# A novel method for heterocyclic amide–thioamide transformations

**DOI:** 10.3762/bjoc.13.20

**Published:** 2017-01-26

**Authors:** Walid Fathalla, Ibrahim A I Ali, Pavel Pazdera

**Affiliations:** 1 Physics and Math. Engineering Dept., Faculty of Engineering, Port-Said University, Port Said, Egypt; 2 Department of Chemistry, Faculty of Science, Suez Canal University, Ismailia, Egypt; 3 Centre for Syntheses at Sustainable Conditions and Their Management, Faculty of Science, Masaryk University, Brno, Czech Republic

**Keywords:** heterocyclic amides, heterocyclic thioamides, *N*-cyclohexyl dithiocarbamate cyclohexylammonium salt, novel thiating agent, thiation

## Abstract

In this paper, we introduce a novel and convenient method for the transformation of heterocyclic amides into heteocyclic thioamides. A two-step approach was applied for this transformation: Firstly, we applied a chlorination of the heterocyclic amides to afford the corresponding chloroheterocycles. Secondly, the chloroherocycles and *N*-cyclohexyl dithiocarbamate cyclohexylammonium salt were heated in chloroform for 12 h at 61 °C to afford heteocyclic thioamides in excellent yields.

## Introduction

Transforming heterocyclic amides into thioamides is an important task in organic synthesis. Earlier reports for this type of O/S conversions were achieved by several thiating reagents; for instance, Lawesson's reagent (2,4-bis(4-methoxyphenyl)-1,3-dithia-2,4-diphosphetane 2,4-disulfide) [1–3], Berzelius reagent [4–6] (P_4_S_10_), and phosphorus pentasulfide [7] in dry toluene, xylene or pyridine under reflux conditions. A two-step approach for the purpose of thiation of heterocyclic amides attracted our attention: as a first step, we applied a chlorination of heterocyclic amides, followed by thiation via reaction with thiourea on the basis of reagent-promoted desulfurylation of isothiourea under strong basic conditions [8–9]. Aiming to continue our reseach work on the structure modification of functionalized heterocyclic amides and thioamides [10–17], we found it interesting to design a new convenient and simple method for the thiation of heterocyclic amides.

## Results and Discussion

Many synthetic methods related to thiation of heterocyclic amides have been reported to date. Most methods suffer from the employment of expensive specific reagents, high temperature, use of strong basic conditions, ultra-dry solvents, bad smell, low yield, difficulties in work-up procedures or from a narrow substrate scope. Therefore, the development of a more efficient method for the transformation of heterocyclic amides to heterocyclic thioamides gained great attention.

**Scheme 1:**
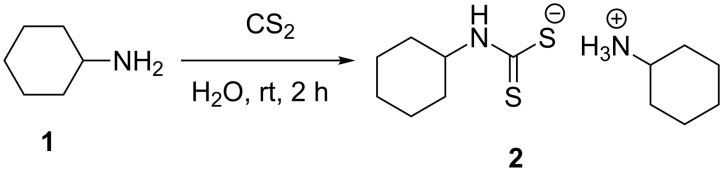
Synthesis of *N*-cyclohexyl dithiocarbamate cyclohexylammonium salt (**2**).

The reaction of three molar equivalents of cyclohexylamine (**1**) with one molar equivalent of carbon disulfide in water typically afforded *N*-cyclohexyl dithiocarbamate cyclohexylammonium salt (**2**) as an excellent new thiating reagent in high yield, Scheme 1.

The structure assignment of the prepared *N*-cyclohexyl dithiocarbamate cyclohexylammonium salt (**2**) is based on ^1^H and ^13^C NMR spectral and physicochemical analysis. The ^1^H NMR spectrum displays a broad singlet signal at 8.01 ppm associated with three NH protons. The ^1^H NMR spectrum also shows three multiplet signals at 4.15–3.95 and 3.05–2.96 and 1.98–0.96 ppm corresponding to two CH and 10 CH_2_ groups, respectively. The ^13^C NMR spectrum of **2** displays signals at δ 212.4, 55.3 and 50.0 ppm associated with (C=S) and two CH groups, respectively. The ^13^C NMR spectrum of **2** also shows signals at 32.3, 30.9, 25.8, 25.5, 25.1, and 24.3 ppm due to cyclohexyl CH_2_ groups.

Heterocyclic amides **A1–13** used in this context were prepared as described in literature expanding simple one-step procedures to multi-step sequential reactions. Quinazoline-4-one (**A1**) [18] was prepared by Niementowski reaction by fusion of anthranilic acid with formamide at 120 °C for 5 h. A number of quinazoline derivatives **A2–A6** [19–21] were prepared via sequential steps starting from easily available carboxylic acid chlorides. The acid chlorides reacted with anthranilic acid to afford benzoxazines, followed by sequential reaction with ammonia to afford the benzanilide derivatives and finally, benzanilides were cyclized by heating in sodium hydroxide solution and gave quinazolines **A2–A6**. Methyl 1,2-dihydro-2-oxoquinoline-4-carboxylate (**A9**) [22–23] was prepared by heating isatine with malonic acid followed by esterification of the produced quinoline carboxylic acid with methanol in the presence of sulfuric acid at 80 °C for 6 h. 4-Arylphthalazin-1(2*H*)-ones **A7** and **A8** [24–25] were prepared by Friedel–Crafts acylation reaction of *N*-aminophthalimide with either benzene or toluene in the presence of AlCl_3_, respectively. A number of quinoxalin-2-one derivatives **A10–13** [26–29] were prepared by the reaction of *o*-phenylenediamine with oxoacids or oxoesters either in HCl solution or in ethanol.

Heterocyclic amides **A1–9** were heated with POCl_3_ for 2–5 h as reported in literature to afford the respective chloroheterocycles [30–37] **B1–9** and **13** and were purified using flash column chromatography; petroleum ether (60–80)/ethyl acetate (9:1) as an eluent. Best results for the preparation of chloroquinoxalinones **B11** and **B12** [38–39] were achieved by dropwise addition of *N*,*N*-dimethylaniline to a stirred cold solution of quinoxalinones **A11** and **A12** and POCl_3_, the reaction mixture was refluxed for 15 minutes.

**Scheme 2:**
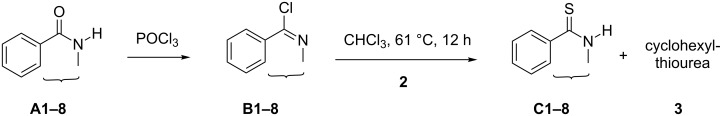
The two-step thiation of quinazolin-4-one **A1–6** and phthalazin-1-ones **A7** and **A8**.

Thus, *N*-cyclohexyl dithiocarbamate cyclohexylammonium salt (**2**) was added to 4-chloro-2-phenylquinazoline (**B2**) solution in CHCl_3,_ the reaction mixture was heated at 61 °C for 12 h. The reaction mixture was evaporated and poured in ethanol to give bright yellow crystals as only isolated product, identified as 2-phenylquinazoline-4(3*H*)-thione (**C2**). The filtrate was once again evaporated and crystalized from ethanol/water to give dicyclohexylthiourea (**3**, Scheme 2). We have extended the scope of this process to involve the transformation of a number of heterocyclic amides; quinazolin-4(3*H*)-one (**A1**), 2-substituted quinazolin-4(3*H*)-one **A3–A6** and 4-subsituted phthalazin-1(2*H*)-ones **A7** and **A8** into the corresponding heterocyclic thioamides **C1** and **C3–C8**, respectively (Scheme 2, Table 1 and Table 2).

**Table 1:** Synthesis of quinazolin-4-thiones^a^.

No.	heterocyclic amide **A**	chloro- heterocycles **B**	heterocyclic thioamide **C**	Yield^b^ %

1	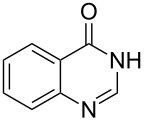 **A1**	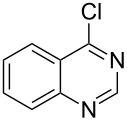 **B1**	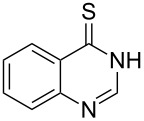 **C1**	76%
2	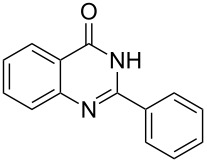 **A2**	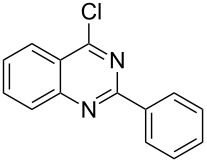 **B2**	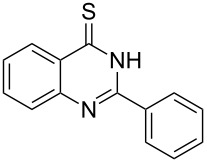 **C2**	92%
3	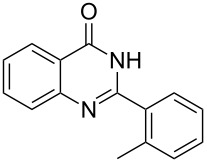 **A3**	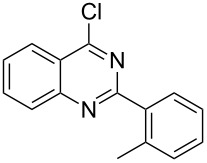 **B3**	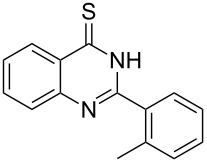 **C3**	84%
4	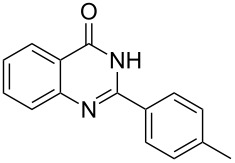 **A4**	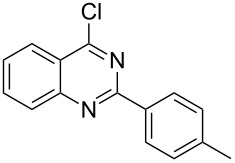 **B4**	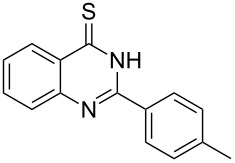 **C4**	89%
5	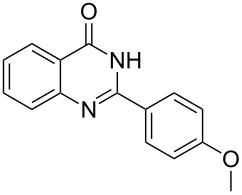 **A5**	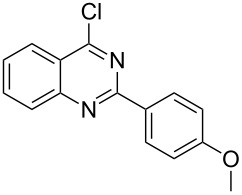 **B5**	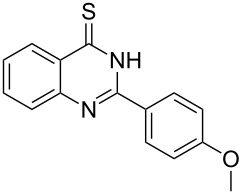 **C5**	95%
6	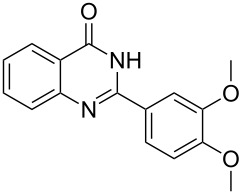 **A6**	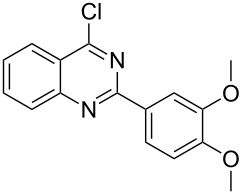 **B6**	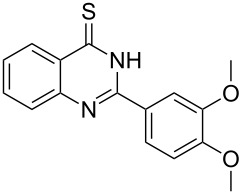 **C6**	81%

^a^Reaction conditions: chloroheterocycles (20 mmol) and *N*-cyclohexyl dithiocarbamate cyclohexylammonium salt (**2**, 20 mmol) were heated in CHCl_3_ (25 mL) at 61 °C for 12 h. ^b^Yields refer to isolated pure product of the reaction from **B** to **C**.

**Table 2:** Synthesis of phthalizin-1-thiones **C7** and **C8**^a^.

No.	heterocyclic amide **A**	chloro- heterocycles **B**	heterocyclic thioamide **C**	Yield^b^ %

7	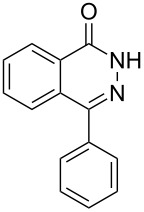 **A7**	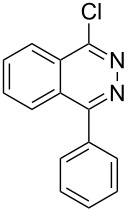 **B7**	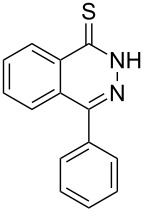 **C7**	91%
8	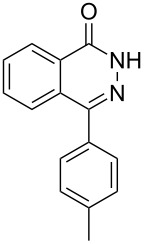 **A8**	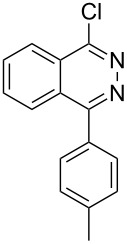 **B8**	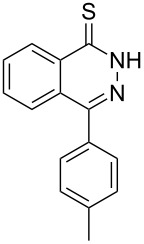 **C8**	78%

^a^Reaction conditions as described before. ^b^Yields refer to isolated pure product of the reaction from **B** to **C**.

**Scheme 3:**
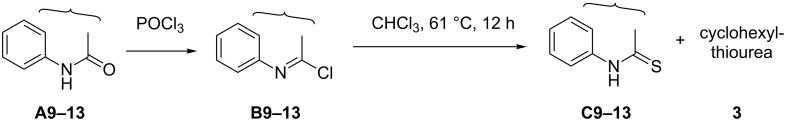
Thiation of quinoline **A9** and quinoxalinone **A10–13**.

The *N*-cyclohexyl dithiocarbamate cyclohexylammonium salt (**2**) has been found to be an excellent reagent for thiation of heterocyclic amides into thioamides at position 4, Scheme 2, Table 1 and Table 2. We have extended the scope of this thiation process to involve heterocyclic amides at positions 2 and 3. Thus, methyl 1,2-dihydro-2-oxoquinoline-4-carboxylate (**A9**) and 3-substituted quinoxalin-2(1*H*)-ones **A10–13** reacted similarly with phosphorous oxychloride to afford the chloro derivatives **B9–13** which were subsequently converted into the corresponding thioamides **C9–13** by the reaction with *N*-cyclohexyl dithiocarbamate cyclohexylammonium salt (**2**) in CHCl_3_ under reflux conditions for 12 h (Scheme 3, Table 3).

**Table 3:** Synthesis of quinolin-2-thiones **C9** and quinoxalin-2-thiones **C10–C13**^a^.

No.	heterocyclic amide **A**	chloro- heterocycles **B**	heterocyclic thioamide **C**	Yield^b^ %

9	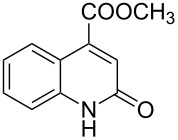 **A9**	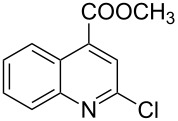 **B9**	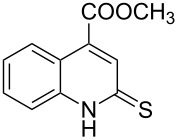 **C9**	76%
10	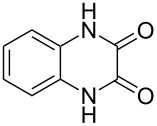 **A10**	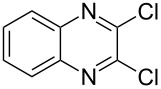 **B10**	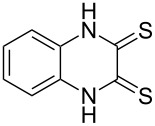 **C10**	69%
11	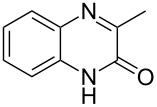 **A11**	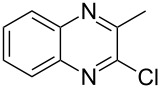 **B11**	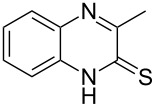 **C11**	83%
12	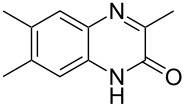 **A12**	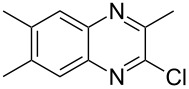 **B12**	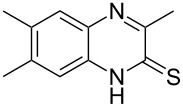 **C12**	72%
13	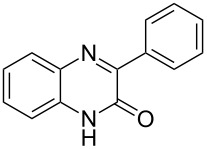 **A13**	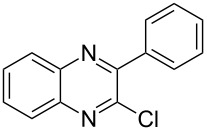 **B13**	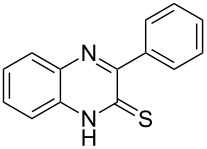 **C13**	91%

^a^Reaction conditions as described before. ^b^Yields refers to isolated pure product of the reaction from **B** to **C**.

The synthetic procedure for the formation of **C1–13** reported herein have the advantage of operational simplicity and availability of both the substrate and the reagents giving a series of very interesting compounds. This method also was adjusted to involve a one-pot strategy starting from heterocyclic amides **A1–13** to directly afford the heterocyclic thioamides **C1–13**. Thus, 2-phenylquinazolin-4(3*H*)-one (**A2**) was heated with phosphorous oxychloride for 2 h. The reaction mixture was evaporated and poured in ice-cold ammonia solution, then extracted with chloroform and dried over sodium sulfate. *N*-cyclohexyl dithiocarbamate cyclohexylammonium salt (**2**) was added to the chloroform solution of chloroquinazoline **B2** and heated at 61 °C for 12 h. The reaction mixture was evapourated and ethanol was added successively to give the desired product **C2**.

The structure assignment of the prepared heterocyclic thioamides **C1–13** is based on ^1^H and ^13^C NMR spectral and physicochemical analyses. The ^1^H NMR spectrum of 2-(4-methoxyphenyl)quinazoline-4(3*H*)-thione (**C5**) gave a broad singlet and a singlet signal at δ 13.71 and 3.87 ppm, associated with NH and OCH_3_ groups, respectively. The significant downfield shift of the NH proton is probably due to intermolecular hydrogen bond interactions of the type NH···S=C. All the isolated thioureas **C1–13** exhibited similar ^1^H NMR spectral patterns with the NH protons at similar chemical shifts and they adopt paired thioamide structures (vide infra). The ^1^H NMR spectrum also shows four doublet and two triplet signals at δ 8.60, 8.19, 7.75, 7.11, 7.88, 7.56, respectively due to eight aromatic protons. The ^13^C NMR spectrum of **C5** displays signals at δ 187.9 and 56.0 ppm due to C=S and OCH_3_, respectively.

**Scheme 4:**
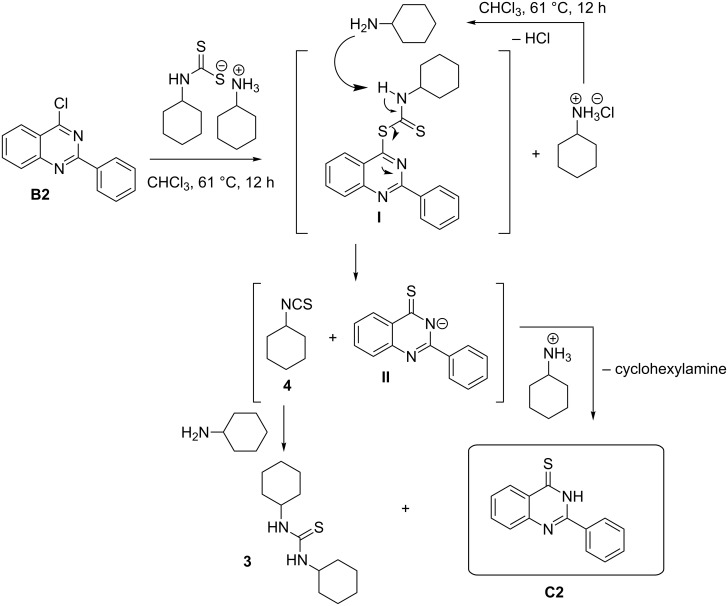
Rational mechanism of the reaction of 4-chloro-2-phenylquinazoline (**B2**) to 2-phenylquinazolin-4(3*H*)-thione.

A mechanistic rationalization for this interesting rearrangement is given in Scheme 4. The reaction of 4-chloro-2-phenylquinazoline (**B2**) with *N*-cyclohexyl dithiocarbamate cyclohexylammonium salt (**2**) in CHCl_3_ at 61 °C for 12 h was principally expected to give 2-phenylquinazolin-4-yl cyclohexylcarbamodithioate (**I**) and cyclohexylamine hydrochloride. Cyclohexylamine hydrochloride under heating conditions will eliminate an HCl molecule forming the free cyclohexylamine base.

Cyclohexylamine will further abstract a proton from **I** followed by electron delocalization and the overall formation of cyclohexyl isothiocyanate (**4**) via C–S bond cleavage and the formation of quinazoline thiol anion **II** having a negative charge concerted on the nitrogen atom. The protonated cyclohexylamine in the previous step will transfer this extra proton to **II** to afford the quinazoline thione **C2**. On the other hand the free cyclohexylamine will add to cyclohexyl isothiocyanate (**4**) to form the thiourea **3**. Similar results were obtained by Furumoto [40], and Sun [41] reported the application of cyanuric chloride (2,4,6-trichloro-1,3,5-triazine, TCT) as a desulfurylation reagent in the synthesis of carbodiimides or alkyl isothiocyanates from thioureas under mild conditions.

## Conclusion

Several synthetic procedures related to thiation of heterocyclic amides have been reported to date. The drawback of the existing methods is the use of expensive specific reagents, high temperature, use of strong basic conditions, ultra-dry solvents, bad smell, low yield, difficulties in work-up procedures or from a narrow substrate scope. In this work, we successfully developed a facile and convenient general method for the transformation of heterocyclic amides into heterocyclic thioamides. Generally, in the proposed technique we transformed heterocyclic amides to chloroheterocyclic compounds by the action of phosphorous oxychloride. Subsequently, chloroheterocyclic derivatives reacted with *N*-cyclohexyl dithiocarbamate cyclohexylammonium salt in chloroform at 61 °C for 12 h to finally afford the heterocyclic thioamides in excellent yields. Furthermore, this method is advantageous over existing methods in the matter of simplicity of the work-up procedure, higher yield, odorless, lower reaction temperature and finally the availability of both precursors and reagent.

## Experimental

### General procedures

Solvents were purified and dried by standard procedures. The boiling range of the petroleum ether used was 40–60 °C. Thin-layer chromatography (TLC): silica gel 60 F_254_ plastic plates (E. Merck, layer thickness 0.2 mm) detected by UV absorption. Elemental analyses were performed on a Flash EA-1112 instrument at the Microanalytical laboratory, Faculty of Science, Suez Canal University, Ismailia, Egypt. Melting points were determined on a Büchi 510 melting-point apparatus and the values are uncorrected. ^1^H and ^13^C NMR spectra were recorded at 300 MHz and 75.5 MHz, respectively (Bruker AC 300) in CDCl_3_ and DMSO solution with tetramethylsilane as an internal standard. The NMR analyses were performed at the Organic Chemistry Department Masaryk University, Brno, Czech Republic. Compounds **A1–13** and **B1–13** were obtained by published methods [18–39], and their melting points and ^1^H and ^13^C NMR spectra corresponded to those given in the literature.

**General method for the preparation of thiating reagent** ***N*-cyclohexyl dithiocarbamate cyclohexylammonium salt (2).** To a mixture of freshly distilled cyclohexylamine (60 mmol) and water (50 mL) was added carbon disulfide (21 mmol) dropwise. The reaction mixture was stirred at room temperature for 2 h. The white solid obtained was filtered, washed with water, dried and crystalized from ethanol to provide the pure product. Yield 98% (ethanol 95%) white crystals, mp 188–189 °C; ^1^H NMR (300 MHz, DMSO-*d*_6_) δ 8.01 (bs, 3H, 3NH), 4.15–3.95 (m, 1H, CH), 3.05–2.96 (m, 1H, CH), 1.98–0.96 (20H, m, 10CH_2_); ^13^C NMR (75.0 MHz, DMSO-*d*_6_) δ 212.4 (C=S), 55.3 (CH), 50.0 (CH), 32.3 (2CH_2_), 30.9 (2CH_2_), 25.8 (CH_2_), 25.5 (2CH_2_), 25.1 (CH_2_), 24.3 (2CH_2_); anal. calcd for C_13_H_26_N_2_S_2_ (274.2): C, 56.56; H, 9.43; N, 10.09; found: C, 56.88; H, 9.55; N, 10.21.

### General method for the preparation of heterocyclic thioamides

**Method A.** To a solution of chloroheterocycles (2.5 mmol) in CHCl_3_ (25 mL) was added (0.69 g, 2.5 mmol) of *N*-cyclohexyl dithiocarbamate cyclohexylammonium salt. The reaction mixture was refluxed at 61 °C for 12 h. The reaction mixture was evaporated under reduced pressure and 25 mL of ethanol was added to the solid residue. The yellowish–orange precipitate was filtered to give the desired product. The crude compounds were pure enough for analytical purposes. Purification of products for analysis was achieved by crystallization from the appropriate solvent; chromatographed with the appropriate eluent or by repeated dissolution in KOH and reprecipitation by acetic acid. The filtrate was evaporated once again and the solid obtained was crystalized from ethanol water to give symmetrical dicyclohexylthiourea (**3**).

**Method B.** To a cold solution of heterocyclic amide (2.5 mmol) in POCl_3_ (25 mL) was added dimethylaniline (2.5 mmol). The reaction mixture was stirred under reflux (100–105 °C) for 1.5–2 h. The excess POCl_3_ was removed under reduced pressure. The residue was poured into a mixture of chloroform (50 mL), ice water (80 mL) and ammonia (5 mL). The chloroform layer was separated, dried over Na_2_SO_4_ and filtered. To this chloroform solution of the in situ generated chloroheterocycles was added (0.69 g, 2.5 mmol) of *N*-cyclohexyl dithiocarbamate cyclohexylammonium salt. The reaction mixture was refluxed at 61 °C for 12 h. The reaction mixture was evaporated under reduced pressure and 25 mL of ethanol was added to the solid residue. The yellowish–orange precipitate was filtered to give the desired product. The crude compounds were pure enough for analytical purposes. Purification of products for analysis was achieved by crystallization from the appropriate solvent; chromatographed with the appropriate eluent or by repeated dissolution in KOH and reprecipitation by acetic acid.

**Dicyclohexylthiourea (3)** [42]: Yield 65% (ethanol 95%–H_2_O) white crystals, mp 180–181 °C; ^1^H NMR (300 MHz, DMSO-*d*_6_) δ 7.05 (bs, 2H, NH), 4.05–3.89 (m, 2H, 2CH), 1.87–1.52 (m, 10H, 5CH_2_) 1.29–1.12 (m, 10H, 5CH_2_); ^13^C NMR (75.0 MHz, DMSO-*d*_6_) δ 180.5 (C=S), 51.9 (CH), 32.8 (2CH_2_), 25.7 (2CH_2_), 25.0 (CH_2_); anal. calcd for C_13_H_24_N_2_S (240.2): C, 64.95; H, 10.06; N, 11.65; found: C, 64.82; H, 10.01; N, 11.46.

**Quinazoline-4(3*H*)-thione (C1)** [43]: Yield 76% (H_2_O) yellow crystals, mp 320–321 °C; ^1^H NMR (300 MHz, DMSO-*d*_6_) δ 13.83 (bs, 1H, NH), 8.55–7.28 (m, 5H, ArH); ^13^C NMR (75.0 MHz, DMSO-*d*_6_) δ 186.2 (C=S), 144.8 (C Ar), 144.2 (CHAr), 135.7 (CHAr), 129.7 (CHAr), 129.4 (C Ar), 128.7 (CHAr), 128.5 (CHAr); anal. calcd for C_8_H_6_N_2_S (162.0): C, 59.23; H, 3.73; N, 17.27; found: C, 59.17; H, 3.69; N, 17.15.

**2-Phenylquinazoline-4(3*H*)-thione (C2)** [44]: Yield 92% (ethanol 95%–DMF) yellow crystals, mp 222–223 °C; ^1^H NMR (300 MHz, DMSO-*d*_6_) δ 13.75 (bs, 1H, NH), 8.63 (d, *J* = 8.0 Hz, 1H, ArH), 8.17 (d, *J* = 8.0 Hz, 2H, ArH), 7.93–7.89 (m, 3H, ArH), 7.82–7.57 (m, 3H, ArH); ^13^C NMR (75.0 MHz, DMSO-*d*_6_) δ 188.5 (C=S), 152.1 (C Ar), 144.8 (C Ar), 135.8 (CHAr), 132.8 (C Ar), 131.9 (CHAr), 129.8 (CHAr), 128.9 (CHAr), 128.6 (CHAr), 128.4 (CHAr), 128.1 (C Ar); anal. calcd for C_14_H_10_N_2_S (238.1): C, 70.56; H, 4.23; N, 11.76; found: C, 70.48; H, 4.16; N, 11.49.

**2-*o*-Tolylquinazoline-4(3*H*)-thione (C3)** [45]: Yield 84% (ethanol 95%–DMF) yellow crystals, mp 183–184 °C; ^1^H NMR (300 MHz, DMSO-*d*_6_) δ 13.97 (bs, 1H, NH), 8.65 (d, *J* = 8.0 Hz, 1H, ArH), 7.96 (t, *J* = 8.0 Hz, 1H, ArH), 7.75 (d, *J* = 8.0 Hz, 1H, ArH), 7.66–7.35 (m, 5H, ArH), 2.39 (s, 3H, CH_3_); ^13^C NMR (75.0 MHz, DMSO-*d*_6_) δ 187.7 (C=S), 155.5 (C Ar), 144.7 (C Ar), 136.8 (C Ar), 135.8 (CHAr), 134.0 (CHAr), 130.9 (C Ar), 130.6 (CHAr), 130.0 (CHAr), 129.7 (CHAr), 128.6 (CHAr), 128.0 (CHAr), 126.1 (C Ar), 19.9 (CH_3_); anal. calcd for C_15_H_12_N_2_S (252.1): C, 71.40; H, 4.79; N, 11.10; found: C, 71.21; H, 4.65; N, 10.94.

**2-*p*-Tolylquinazoline-4(3*H*)-thione (C4)** [46]: Yield 89% (ethanol 95%–DMF) yellow crystals, mp 218–219 °C; ^1^H NMR (300 MHz, DMSO-*d*_6_) δ 13.78 (bs, 1H, NH), 8.62 (d, *J* = 8.0 Hz, 1H, ArH), 8.10 (d, *J* = 8.0 Hz, 2H, ArH), 7.93–7.76 (m, 2H, ArH), 7.58 (t, *J* = 8.0 Hz, 1H, ArH), 7.37 (d, *J* = 8.0 Hz, 2H, ArH), 2.41 (s, 3H, CH_3_); ^13^C NMR (75.0 MHz, DMSO-*d*_6_) δ 187.9 (C=S), 151.9 (C Ar), 149.3 (C Ar), 142.1 (C Ar), 135.9 (CHAr), 130.4 (C Ar), 129.8 (CHAr), 129.7 (CHAr), 129.6 (CHAr), 128.8 (CHAr), 126.3 (CHAr), 128.0 (CHAr), 126.4 (C Ar), 21.5 (CH_3_); anal. calcd for C_15_H_12_N_2_S (252.1): C, 71.40; H, 4.79; N, 11.10; found: C, 71.28; H, 4.61; N, 11.84.

The yield, ^1^H, ^13^C NMR spectral data and physicochemical analysis of other prepared thioamides (**C5**–**C13**) are presented in Supporting Information File 1.

## Supporting Information

File 1 Additional experimental and analytical data.
