# Supplementary material for: A novel method for heterocyclic amide–thioamide transformations
Source: Beilstein J Org Chem. 2017 Jan 26;13:174–81. doi: 10.3762/bjoc.13.20 (PMC5302013; doi:10.3762/bjoc.13.20)
Supplement: File 1 — Additional experimental and analytical data. [file Beilstein_J_Org_Chem-13-174-s001.pdf]

Supporting Information

for

A novel method for heterocyclic amide–thioamide transformations

Walid Fathalla[1]*, Ibrahim A. I. Ali[2] and Pavel Pazdera[3]

Address: [1]Physics and Math. Engineering Dept., Faculty of Engineering, Port-Said University, Port Said, Egypt, [2]Department of Chemistry, Faculty of Science, Suez Canal University, Ismailia, Egypt and [3]Centre for Syntheses at Sustainable Conditions and Their Management, Faculty of Science, Masaryk University, Brno, Czech Republic

Email: Walid Fathalla - walid3369@yahoo.com

*Corresponding author

Additional experimental and analytical data

General method for the preparation of heterocyclic thioamides

Method A.

To a solution of chloroheterocycles (2.5 mmol) in $CHCl_3$ (25 mL) was added (0.69 g, 2.5 mmol) of *N*-cyclohexyl dithiocarbamate cyclohexylammonium salt. The reaction mixture was refluxed at 61 °C for 12 h. The reaction mixture was evaporated under reduced pressure and 25 mL of ethanol was added to the solid residue. The yellowish –orange precipitate was filtered to give the desired product. The crude compounds were pure enough for analytical purposes. Purification of products for analysis was achieved by crystallization from the appropriate solvent; chromatographed with the appropriate eluent or by repeated dissolution in KOH and reprecipitation by acetic acid. The filtrate was evaporated once again and the solid obtained was crystalized from ethanol water to give symmetrical dicyclohexyl thiourea (**3**).

Method B.

To a cold solution of heterocyclic amide (2.5 mmol) in $POCl_3$ (25 mL) was added dimethyl aniline 2.5 mmol. The reaction mixture was stirred under reflux (100–105 °C) for 1.5–2 h. The excess $POCl_3$ was removed under reduced pressure. The residue was poured into a mixture of chloroform (50 mL), ice water (80 mL) and ammonia (5 mL) The chloroform layer was separated, dried over Na_2SO_4 and filtered. To this chloroform solution of the insitu generated chloroheterocycles was added (0.69g, 2.5 mmol) of *N*-cyclohexyl dithiocarbamate cyclohexylammonium salt. The reaction mixture was refluxed at 61 °C for 12 h. The reaction mixture was evapourated under reduced pressure and 25 mL of ethanol was added to the solid residue. The yellowish–orange precipitate was filtered to give the desired product. The crude compounds were pure enough for analytical purposes. Purification of products for analysis was

achieved by crystallization from the appropriate solvent; chromatographed with the appropriate eluent or by repeated dissolution in KOH and reprecipitation by acetic acid.

2-(4-Methoxyphenyl)quinazoline-4(3*H*)-thione (C5) [1,2].

Yield 95% (ethanol 95%-DMF) yellow crystals, mp 199-200°C. ^{1}H NMR spectrum, (300 MHz, DMSO), δ, ppm (*J*, Hz): 13.71 (1H, bs, NH); 8.60 (1H, d, *J* = 8.0, ArH); 8.19 (2H, d, *J* = 8.0, ArH); 7.88 (1H, t, *J* = 8.0, ArH); 7.75 (1H, d, *J* = 8.0, ArH); 7.56 (1H, t, *J* = 8.0, ArH); 7.11 (2H, d, *J* = 8.0, ArH); 3.87 (3H, s, CH_3). ^{13}C NMR spectrum, (75.0 MHz, DMSO), δ, ppm: 187.9 (C=S); 162.5 (C Ar); 151.5 (C Ar); 149.3 (C Ar); 135.9 (CHAr); 130.7 (CHAr); 129.8 (C Ar); 128.5 (CHAr); 128.0 (CHAr); 127.8 (CHAr); 124.6 (C Ar); 122.2 (CHAr); 114.4 (CHAr); 56.0 (CH_3). Found, %: C, 66.87; H, 4.39; N, 10.27; S, 11.95. For $C_{15}H_{12}N_2OS$ (268.1). Calculated, %: C, 67.14; H, 4.51; N, 10.44.

2-(3,4-Dimethoxyphenyl)quinazoline-4(3*H*)-thione (C6).

Yield 81% (ethanol 95%-DMF) yellow crystals, mp 218-219°C. ^{1}H NMR spectrum, (300 MHz, DMSO), δ, ppm (*J*, Hz): 13.73 (1H, bs, NH); 8.61 (1H, d, *J* = 8.0, ArH); 7.89–7.70 (4H, m, ArH); 7.56 (1H, t, *J* = 8.0, ArH); 7.13 (1H, d, *J* = 8.0, ArH); 3.91 (3H, s, CH_3); 3.86 (3H, s, CH_3). ^{13}C NMR spectrum, (75.0 MHz, DMSO), δ, ppm: 187.9 (C=S); 152.3 (C Ar); 151.4(C Ar); 148.9 (C Ar); 135.9 (C Ar); 129.8 (C Ar); 128.0 (CHAr); 127.8 (CHAr); 124.5 (C Ar); 122.4 (C Ar); 111.9 (CHAr); 111.8 (CHAr); 56.3 (CH_3); 56.2 (CH_3). Found, %: C, 64.28; H, 4.63; N, 9.24. For $C_{16}H_{14}N_2O_2S$ (298 .1). Calculated, %: C, 64.41; H, 4.73; N, 9.39.

4-Phenylphthalazine-1(2*H*)-thione (C7) [3].

Yield 91% (toluene) yellow crystals, mp 212-213°C. ^{1}H NMR spectrum, (300 MHz, DMSO), δ, ppm (*J*, Hz): 14.55 (1H, bs, NH); 8.85–8.79 (1H, m, ArH); 7.99–7.96 (2H, m, ArH); 7.77–7.75 (1H, m, ArH); 7.62–7.57 (5H, m, ArH). ^{13}C NMR spectrum, (75.0 MHz, DMSO), δ, ppm: 180.9 (C=S); 152.2 (C Ar); 135.0 (CHAr); 134.7 (C Ar); 134.2 (C Ar); 133.4 (CHAr); 130.3 (CHAr); 129.9 (CHAr); 129.1 (CHAr); 127.5 (CHAr); 124.2 (C Ar). Found, %: C, 70.45; H, 4.14; N, 11.70. For $C_{14}H_{10}N_2S$ (238.1). Calculated, %: C, 70.56; H, 4.23; N, 11.76.

4-*p*-Tolylphthalazine-1(2*H*)-thione (C8) [4].

Yield 78% (toluene) yellow crystals, mp 232-233 °C. ^{1}H NMR spectrum, (300 MHz, DMSO), δ, ppm (*J*, Hz): 14.45 (1H, bs, NH); 8.84–8.81 (1H, m, ArH); 7.98–7.95 (2H, m, ArH); 7.77–7.76 (1H, m, ArH); 7.52 (2H, d, *J* = 8.0, ArH); 7.39 (2H, d, *J* = 8.0, ArH); 2.42 (3H, s, CH_3). ^{13}C NMR spectrum, (75.0 MHz, DMSO), δ, ppm: 180.7 (C=S); 152.2 (C Ar); 139.5 (C Ar); 134.9 (CHAr); 134.2 (C Ar); 133.3 (CHAr); 131.9 (C Ar); 130.3 (CHAr); 129.8 (CHAr); 129.6 (CHAr); 127.6 (CHAr); 124.3 (C Ar); 21.4 (CH_3). Found, %: C, 71.27; H, 4.63; N, 11.01. For $C_{15}H_{12}N_2S$ (252.1). Calculated, %: C, 71.40; H, 4.79; N, 11.10.

Methyl 1,2-dihydro-2-thioxoquinoline-4-carboxylate (C9) [5].

Yield 76% orange crystals (ethanol 95%), mp 198-199°C. ^{1}H NMR spectrum, (300 MHz, DMSO), δ, ppm (*J*, Hz): 12.92 (1H, bs, NH); 8.47 (1H, d, *J* = 8.0, ArH); 7.94 (1H, s, *J* = 8.0, CH); 7.63 (2H, d, *J* = 8.0, ArH); 7.46–7.38 (1H, m, ArH); 4.03 (3H, s, CH_3). ^{13}C NMR spectrum, (75.0 MHz, DMSO), δ, ppm: 180.4 (C=S ester); 165.4 (C=O); 140.4 (C Ar); 139.7 (CHAr); 133.7 (CHAr); 131.7 (CHAr); 126.6 (CHAr);

125.5 (CHAr); 120.0 (C Ar); 116.3 (C Ar); 53.0 (CH_3). Found, %: C, 60.17; H, 4.05; N, 6.28. For $C_{11}H_9NO_2S$ (219.0). Calculated, %: C, 60.26; H, 4.14; N, 6.39.

Quinoxaline-2,3(1*H*,4*H*)-dithione (C10) [6].

Yield 69% (purified for analysis by repeated dissolving in KOH and reprecipitation by acetic acid) brown powder, mp 290-295 decomp. °C. ^{1}H NMR spectrum, (300 MHz, DMSO), δ, ppm (*J*, Hz): 14.18 (2H, bs, 2NH); 7.24 (2H, d, *J* = 8.0, ArH); 7.11 (2H, d, *J* = 8.0, ArH). ^{13}C NMR spectrum, (75.0 MHz, DMSO), δ, ppm: 179.8 (2C=S); 128.3 (2C Ar); 126.0 (2CHAr); 116.0 (2CHAr). Found, %: C, 49.35; H, 3.08; N, 14.38. For $C_8H_6N_2S_2$ (194.0). Calculated, %: C, 49.46; H, 3.11; N, 14.42.

3-Methylquinoxaline-2(1*H*)-thione (C11) [7,8].

Yield 83% (chromatographed with benzene/AcOEt 10: 1) light brown powder, mp 250-251 °C. ^{1}H NMR spectrum, (300 MHz, DMSO), δ, ppm (*J*, Hz): 14.32 (1H, bs, NH); 7.78–7.76 (1H, m, ArH); 7.59–7.52 (2H, m, ArH); 7.44–7.38 (1H, m, ArH); 2.63 (3H, s, CH_3). ^{13}C NMR spectrum, (75.0 MHz, DMSO), δ, ppm: 175.5 (C=S); 161.8 (C Ar); 135.3 (C Ar); 132.1 (C Ar); 130.5 (CHAr); 128.5 (CHAr); 126.0 (CHAr); 116.1 (CHAr); 25.1 (CH_3). Found, %: C, 61.11; H, 4.34; N, 15.66. For $C_9H_8N_2S$ (176.0). Calculated, %: C, 61.34; H, 4.58; N, 15.90.

3,6,7-Trimethylquinoxaline-2(1*H*)-thione (C12).

Yield 72% (chromatographed with benzene/AcOEt 10: 1) yellow powder, mp 264-265°C. ^{1}H NMR spectrum, (300 MHz, DMSO), δ, ppm (*J*, Hz): 14.16 (1H, bs, NH); 7.48 (1H, s, ArH); 7.25 (1H, s, ArH); 2.60 (3H, s, CH_3); 2.29 (6H, s, 2CH_3). ^{13}C NMR spectrum, (75.0 MHz, DMSO), δ, ppm: 179.1 (C=S); 160.6 (C Ar); 140.3 (C Ar);

135.2 (C Ar); 134.1 (CHAr); 130.3 (CHAr); 128.1 (CHAr); 115.8 (CHAr); 25.0 (CH_3); 20.3 (CH_3); 19.7 (CH_3). Found, %: C, 64.60; H, 5.84; N, 13.64. For $C_{11}H_{12}N_2S$ (204.1). Calculated, %: C, 64.67; H, 5.92; N, 13.71.

3-Phenylquinoxaline-2(1*H*)-thione (C13) [8,9].

Yield 91% (chromatographed with benzene/AcOEt 10: 1) yellow powder, mp 224-225°C. ^{1}H NMR spectrum, (300 MHz, DMSO), δ, ppm (*J*, Hz): 14.56 (1H, bs, NH); 8.48-8.37 (1H, m, ArH); 8.18-8.01 (2H, m, ArH); 7.85-7.78 (1H, m, ArH); 7.41–7.33 (5H, m, ArH). Found, %: C, 70.13; H, 3.84; N, 11.29. For $C_{14}H_{10}N_2S$ (236.1). Calculated, %: C, 70.56; H, 4.23; N, 11.76.

References

1. Hanusek J.; Hejtmankova L.; Kubicova L.; Sedlak M. *Molecules* **2001,** *6*, 323.
2. Zheng Y.,; Bian M.; Deng X.; Wang S.; Quan Z. *Archiv der Pharmazie* **2013**, *346*, 119.
3. Hemdan M. M.; Taha S. M.; Gabr A. M.; Elkady M. Y., *Journal of Chemical Research* **2010**, 102.
4. Moustafa A. H.; El Sayed H. A.; Abd El-Hady R. A.; Haikal A. Z.; El-Hashash M. *J. Heterocyclic Chem*. **2016,** *53*, 789.
5. Nakano S.; Yoshida T.; Taniguchi H.; Yashuki M. *Chem. Pharm. Bull*. **1980,** *28***,** 49.
6. Morrison D. C.; Furst *A. J. Org. Chem.* **1956**, *21*(4) 470.
7. Caleb, A. A.; Ballo, D.; Rachid, B.; Hancali A.; Mostapha, B.; Abdelfettah, Z.; Rajae E.; El Mokhtar E. *Arkivoc* **2011**, 217.
8. Nishio,T. *Helv. Chem. Acta*, **1992,** *75*, 487.
9. Lu Z. E; Sun D. Q.; Xu T. L.; Wan J.; Xu, L. C.; Chen K. Q. *Organic Preparations and Procedures International* **1992**, *24*, 358.
